# Chromosome-level genome assembly and annotation of rare and endangered tropical bivalve, *Tridacna crocea*

**DOI:** 10.1038/s41597-024-03014-8

**Published:** 2024-02-10

**Authors:** Jun Li, Haitao Ma, Yanpin Qin, Zhen Zhao, Yongchao Niu, Jianmin Lian, Jiang Li, Zohaib Noor, Shuming Guo, Ziniu Yu, Yuehuan Zhang

**Affiliations:** 1 Key Laboratory of Tropical Marine Bio-resources and Ecology, Guangdong Provincial Key Laboratory of Applied Marine Biology, Innovation Academy of South China Sea Ecology and Environmental Engineering, South China Sea Institute of Oceanology, Chinese Academy of Science, Guangzhou, 510301 China; 2 Hainan Key Laboratory of Tropical Marine Biotechnology, Hainan Sanya Marine Ecosystem National Observation and Research Station, Sanya, 572024 China; 3 Daya Bay Marine Biology Research Station, Chinese Academy of Sciences, Shenzhen, 518124 China; 4 Southern Marine Science and Engineering Guangdong Laboratory (Zhuhai), Zhuhai, 519015 China; 5 Biozeron Shenzhen, Inc, Shenzhen, 518000 China; 6 University of Chinese Academy of Sciences, Beijing, 100049 China

**Keywords:** Genome, Marine biology

## Abstract

*Tridacna crocea* is an ecologically important marine bivalve inhabiting tropical coral reef waters. High quality and available genomic resources will help us understand the population structure and genetic diversity of giant clams. This study reports a high-quality chromosome-scale *T. crocea* genome sequence of 1.30 Gb, with a scaffold N50 and contig N50 of 56.38 Mb and 1.29 Mb, respectively, which was assembled by combining PacBio long reads and Hi-C sequencing data. Repetitive sequences cover 71.60% of the total length, and a total of 25,440 protein-coding genes were annotated. A total of 1,963 non-coding RNA (ncRNA) were determined in the *T. crocea* genome, including 62 micro RNA (miRNA), 58 small nuclear RNA (snRNA), 83 ribosomal RNA (rRNA), and 1,760 transfer RNA (tRNA). Phylogenetic analysis revealed that giant clams diverged from oyster about 505.7 Mya during the evolution of bivalves. The genome assembly presented here provides valuable genomic resources to enhance our understanding of the genetic diversity and population structure of giant clams.

## Background & Summary

Giant clams are tropical marine shellfish mainly distributed in the Indian Ocean, Western Pacific, and South China Sea. There are twelve species of giant clams, divided into two genera, with10 species in Tridacna and 2 in Hippopus^1^. They play a crucial role in coral reef ecosystems, contributing over 60% of the biomass of coral reef ecosystems^2^. Giant clams support coral reef biodiversity, offer habitats, breeding and feeding grounds to various marine organisms, and have extremely important ecological value^3,4^. Giant clams are hermaphrodites, initially functioning as males and later developing female gonads and functioning both as male and female^5^. To avoid the occurrence of self-fertilization, giant clams first release sperm, and then eggs^6^. Bivalves often form symbiotic associations with bacteria, algae, and other marine fauna^7^. There is a symbiotic relationship between giant clams and zooxanthellae. Unlike intracellular symbiosis in stony corals, the zooxanthellae in clams are intercellular and live within the mantle^8^. The symbionts supply nutrients to the host through photosynthesis. While also obtaining some essential nutrients from the host. Notably, symbionts are not transmitted vertically and must be acquired from the environment during the ontogeny of the second larval stage, veliger^9^. Additionally, some bivalves from deep sea engage in symbiosis with chemosynthetic bacteria, which are the primary producers of deep-sea cold seeps and vents^10^.

Among Tridacna species, *T. crocea* is the smallest, with a maximum shell length of no more than 20 cm, growing at a rate of about 4 cm per year, reaching sexual maturity in 1–2 years^11^. The shell is shallow, with two equal sides and the same shape and size. Despite its slow growth and small size, *T. crocea* is known for its vibrant colors and beautiful appearance, making it valuable in food markets, the aquarium trade markets and tropical coral reef ecosystems^12^. Moreover, its photoautotrophic characteristics contribute to oxygen production, benefiting marine organisms^13^. However, anthropogenic disturbances, such as global warming, habitat destruction and over-harvesting, have led to declining giant clam populations, resulting in giant clams been listed on the IUCN red list (IUCN, 2007).

Despite the ecological importance of giant clams, their genomic features have remained unclear. In fact, previous molecular studies of giant claims have focused on phylogeographical patterns^14,15^, as well as the expression and functional analysis of specific genes^16,17^. Limited transcriptome data are available^18,19^. Recently, a genomic survey and resources for *T. crocea* were conducted, which involved determining the genome size, predicting unique content, and providing partial annotations, and assemblies^20^. The lack of genomic information has been a hindrance to the study of the evolutionary and ecological characteristics of giant clam. Recently, the Pacific Biosciences (PacBio) high-fidelity reads (HiFi) have been successfully applied to various complex species and sex chromosomes, such as cultivated apple (high heterozygous)^21^, cultivated alfalfa (utotetraploid)^22^, and human X chromosome^23^. In the present study, the chromosome-level genome of *T*. *crocea* was analyzed for the first time using PacBio HiFi reads, Phase genomics Proximo Hi-C technologies, and Illumina short-read sequencing. In order to predict the relationship between *T. crocea* and other bivalves, gene prediction, functional annotation and phylogenetic analysis were performed. The genome sequence of the giant clam is an important resource for genetic and breeding studies.

## Methods

### Experimental samples collection and sequencing

*T. crocea* were sampled from a tropical marine biological research station in Sanya, Hainan province. The giant clams were immediately anaesthetized, and muscle was extracted for DNA isolation using the modified cetyltrimethylammonium bromide (CTAB) method. The quality and quantity of genomic DNA were assessed using a NanoDrop 2000 spectrophotometer (Thermo Fisher Scientific) and a Qubit 2.0 fluorometer (Thermo Fisher Scientific). DNA integrity was confirmed using a 0.8% agarose gel.

Three distinct genome libraries were created and sequenced in accordance with the manufacturer’s instructions to produce a chromosome-scale assembly of the giant clam: (i) In accordance with the standard PacBio methodology, PCR-free SMRTbell DNA libraries were created utilizing the BluePippin size selection system. The PacBio Sequel system was used to produce long reads; (ii) Phase Genomic’s Hi-C chromosomal conformation captured reads were prepared with the Proximo Hi-C (Animal) Prep Kit and sequenced; (iii) Purified DNA was sheared using a focused ultrasonicator (Covaris) and then used for 350-bp paired-end library construction with the Next Ultra DNA library prep kit (NEB) for Illumina sequencing, the Illumina NovaSeq. 6000 platform was used to sequence short reads (150 bp in length). RNA was extracted from the giant clam mantle and sequenced on the Illumina NovaSeq platform in order to fully aid gene annotation. To construct a high-quality reference genome for the *Tridacna crocea*, the whole genome sequencing generated ~167 × Pacbio Sequel long reads (218.24 Gb) (Table 1), ~105 × Hi-C reads (136.70 Gb) and ~45 × Illumina paired-end reads (58.50 Gb) (Table 2).

**Table 1:** Statistic of Pacbio whole genome sequencing data.

Library	Subread number	Total bases (Gb)	Average length (bp)	Max Reads Length (bp)
DC13	13,376,796	53.25	3,981	209,166
DC23	16,368,970	67.8	4,142	262,698
DC27	19,474,512	97.19	4,991	260,834
Total	49,220,278	218.24	4,434	262,698

**Table 2:** Statistic of illumina data.

Data	rawReads (M)	Raw Bases (Gb)	Clean Reads (M)	Clean Bases (Gb)	clean Rate (%)	Q20 Rate (%)	Q30 Rate (%)
Hi-C	911,301,806	136.7	888,213,450	129.98	95.09	97.12	92.06
Re-sequencing	389,989,060	58.5	386,035,170	53.97	92.29	95.39	86.61
RNA-seq	462.76	69.41	420.09	63.01	90.79	96.68	88.8

### Genome assembly with Pacbio data and Hi-C data

**Fig. 1:**
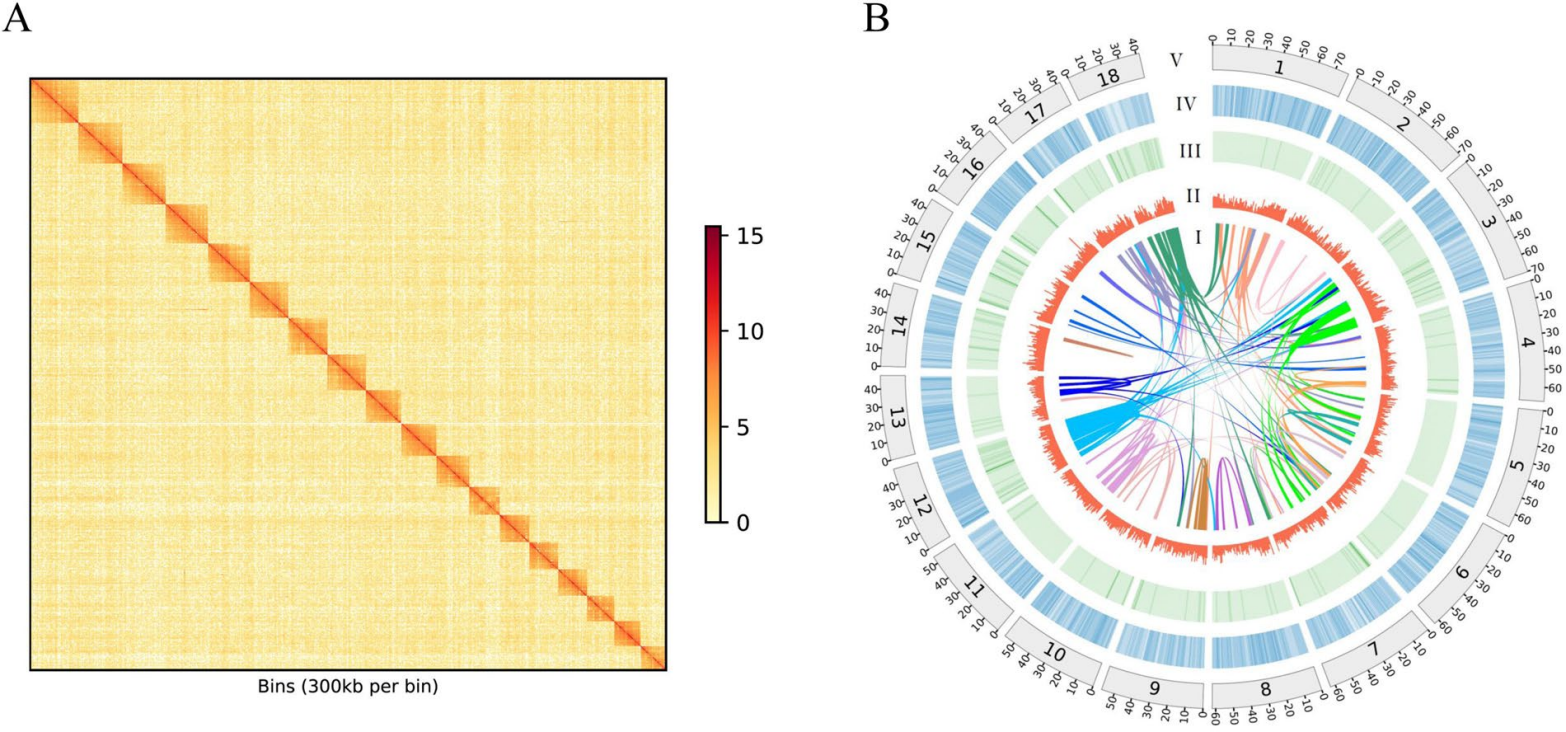
Hi-C contact heatmap and Circos plot illustrate the characterization of *Tridacna crocea* genome, (**A**) Genome-wide analysis of chromatin interactions in the *Tridacna crocea* genome. (**B**) I: Syntenic regions within the *Tridacna crocea* assembly base on homology searches carried out by conducting with MCscan (version 0.8) requiring at least 10 genes per block; II: GC content in non-overlapping 1 Mb windows; III: Percent coverage of TEs in non-overlapping 1 Mb windows; IV: Gene density calculated on the basis of the number of genes in non-overlapping 1 Mb windows; V: The length of scaffolds in the size of Mb.

The Pacbio reads were firstly assembled with Falcon software packages (v2.0.5)^24^ to build the primary contigs and alternate haplotigs (alternative sequences for regions within the primary contigs where heterozygosity was detectable with the long reads). Tool arrow (v2.2.2) as implemented in SMRTlink6.0 (Pacific Biosciences of California, Inc) was used to polish the contigs. The FALCON-Phase software (v0.2.0-beta) was then used to perform a Hi-C-based contigs phasing, resulting in phased, diploid contigs. The chromosome-scale scaffolds were constructed from the phased contigs using Phase Genomics’ Proximo Hi-C genome scaffolding platform^25^. Subsequently, Juicebox (v1.8.8)^26^ was used for a round of polishing to fix minor mistakes in chromosome assignment, ordering, and orientation during chromosomal scaffolding. After a draft set of scaffolds was generated, FALCON-Phase was run again for Hi-C based scaffold phasing. The Illumina sequencing data were further used to improve the assembly by Pilon (v1.22) software^27^. Finally, the Pacbio reads were initially assembled with Falcon software packages, producing an initial contig assembly, then the assembly was integrated with Phase Genomics Hi-C data to orient and order contigs into chromosome-scale scaffolds. About 78.88% of the 1.30 Gb final *Tridacna crocea* assembly was assigned to 18 superscaffolds (Fig. 1), with a scaffold N50 and contig N50 of 56.38 Mb and 1.29 Mb, respectively (Table 3). The length distribution of pacbio long reads indicates the peak length is longer than 4 kb (Fig. S1). This result is consistent with the results of other aquatic animals^28–32^.

**Table 3:** Features of *Tridacna crocea* genome.

Assembly feature	Statistic
Assembly length	1,303,319,575
Contig N50 (bp)	1,291,020
Scaffold N50 (bp)	56,384,102
Number of predicted protein-coding genes	25,440
Repeat content (%)	71.6
Complete BUSCOs (%)	94.2

### Repeat annotation

**Fig. 2:**
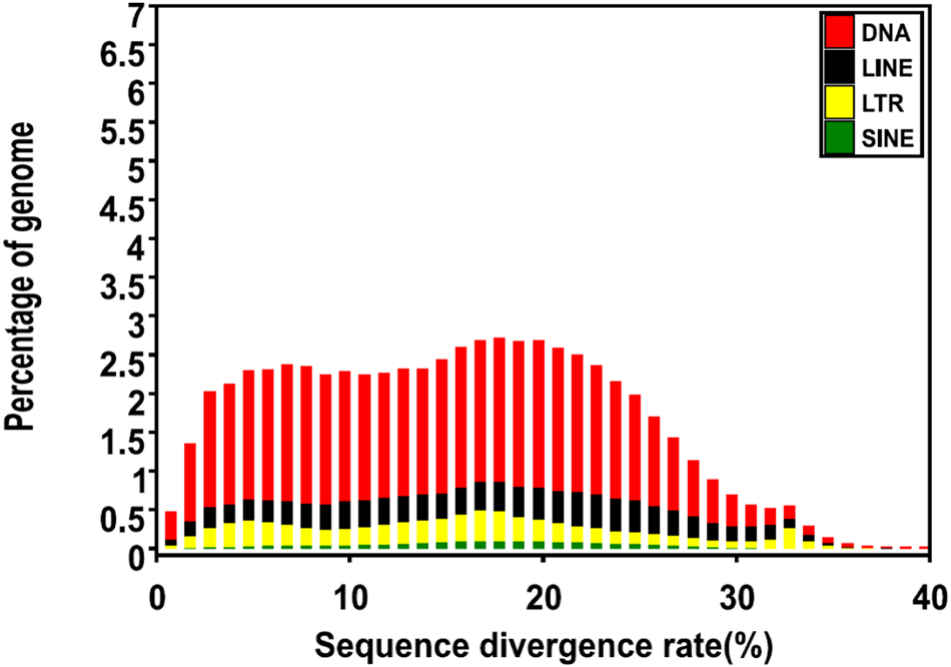
Distribution of divergence rate of each type of TE. The divergence rate was calculated between the identified TE elements in the genome by homology-based method and the consensus sequence in the Repbase.

There are a large number of repeat sequences in the *Tridacna crocea* genome, which can be divided into two categories according to the distribution pattern, namely tandem repeat sequences and interspersed repeat sequences. Tandem repetitive sequences were identified using GMATA^33^ and Tandem Repeats Finder (TRF, version 4.07b)^34^ with default parameters. The interspersed repeat contents of the *Tridacna crocea* genome were identified using two methods, de novo repeat identification and known repeat searching against existing databases. RepeatModeler (v1.0.11) and MITE-hunter^35^ were used to de novo predict repeat sequences in the genome, the homology-based approach involved applying RepeatMasker (version 1.331) (http://www.repeatmasker.org/) and Repbase database^36^ to identify TE repeats in the assembled genome. The results showed that 71.60% of the assembly consisted of repetitive sequences (Table 4, Fig. 2). The proportion of repeat elements was higher than that of close relatives of mollusks, such as *Patinopecten yessoensis* (39%)^37^, *Crassostrea gigas* (43%)^38^ and *Sinonovacula constricta* (40%)^29^, given that repetitive sequences are the main drivers of genome amplification, *T.crocea* presents a larger genome size compared to the three closely related species (Table 5). Among these repetitive sequences, transposable elements (TEs) accounted for 55.83% of the *T. crocea* genome size, with DNA transposons to be the most predominant type (37.68% of the genome size).

**Table 4:** Repeat content in the assembled *Tridacna crocea* genome.

Class	Order	Super family	Number of elements	Percentage of sequence (%)
**Class I**			1,463,284	17.12
	LINE		722,103	8.45
		Unknown	647,388	6.67
		CR1-Zenon	10,831	0.36
		I	13,525	0.43
		RTE-X	19,919	0.53
		L1-Tx1	5,211	0.12
		Other	25,229	0.34
	LTR		591,839	6.92
		Unknown	539,078	5.53
		Pao	6,390	0.17
		Gypsy	26,845	1.01
		DIRS	4,001	0.12
		Other	15,525	0.1
	SINE		149,342	1.75
		tRNA-RTE	70,833	0.94
		Unknown	50,851	0.53
		MIR	19,458	0.22
		Other	8,200	0.06
**Class II**			2,923,451	38.71
	DNA		2,823,422	37.68
		Maverick	16,618	0.44
		Unknown	2,578,864	34.9
		TcMar-Mariner	7,207	0.16
		TcMar-Tc1	13,056	0.24
		hAT-Tip100	44,615	0.51
		P	69,177	0.66
		Other	93,885	0.76
	RC		100,029	1.03
		Helitron	100,029	1.03
**Total TEs**			4,386,735	55.83
**Tandem Repeats**			106,486	0.93
	Tandem repeat		66,402	0.89
	SSR		40,084	0.04
**Simple repeats**			21,650	0.23
**Other**			25,337	0.27
**Unknown**			1,387,494	14.32
**Low complexity**			1,331	0.02
**Total Repeats**			5,929,033	71.6

Note: “Other” refers to a sequence that is classified by softwares but does not belong to any of the above categories, and “Unknown” refers to a sequence that cannot be classified.

**Table 5:** Features of Mollusk assemblies.

Assembly feature	*Tridacna crocea*	*Crassostrea gigas*	*Patinopecten yessoensis*	*Sinonovacula constricta*
Assembly size (Mb)	1,303	647	998	1,220
Contig N50 (kb)	1,291	1,814	38	977
Scaffold N50 (kb)	56,384	58,463	804	65,930
Number of predicted protein-coding genes	25,440	30,724	26,415	28,594
Repeat content (%)	72	43	39	40
Complete BUSCOs (%)	94.2	95.6	94.4	91.9

### Gene prediction and functional annotation

**Fig. 3:**
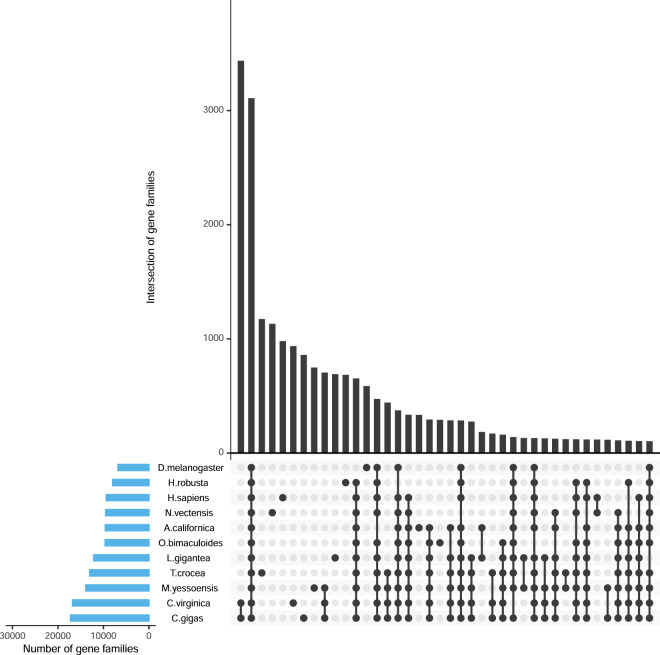
Intersections of gene families between eleven animals (*Tridacna crocea, Aplysia californica, Crassostrea gigas, Crassostrea virginica, Helobdella robusta, Lottia gigantea, Mizuhopecten yessoensis, Octopus bimaculoides, Drosophila melanogaster, Homo sapiens and Nematostella vectensis*). The figure was constructed by UpSetR, in which the rows represent the gene families and the columns represent their intersections. Black filled circle represents part of a given intersection; light gray circle represents not a part of the intersection. Bar chart placed on top of the matrix represents the size of the intersections. A second bar chart represents the size of the each set.

Gene prediction in a repeat-masked genome was performed using reference guided transcriptome assembly, homology search and *ab initio* prediction. By combining transcriptome alignment, homologous protein prediction and *ab initio* prediction. In detail, proteins of four mollusks (*Crassostrea gigas*, *Crassostrea virginica*, *Mizuhopecten yessoensis*, *Octopus bimaculoides*) were downloaded from NCBI DataBase for homolog prediction, GeMoMa^39^ was used to align the homologous peptides to the assembly and then got the gene structure information. For RNAseq-based gene prediction, filtered mRNA-seq reads were aligned to the reference genome using STAR^40^. The transcripts were then assembled using StringTie2^41^ and open reading frames (ORFs) were predicted using PASA^42^. For the de novo prediction, RNA-seq reads were de novo assembled using stringtie and analyzed with PASA to produce a training set. Augustus^43^ with default parameters were then utilized for ab initio gene prediction with the training set. Finally, EVidenceModeler (EVM)^44^ was used to produce an integrated gene set of which gene with TE were removed using TransposonPSI package (http://transposonpsi.sourceforge.net/) and the miscoded genes were further filtered. Untranslated regions (UTRs) and alternative splicing regions were determined using PASA based on RNA-seq assemblies. We retained the longest transcripts for each locus, and regions outside of the ORFs were designated UTRs. We predicted 25,440 protein-coding genes with an average gene length of 25,946 bp and an average 8.43 exons per gene. Functional annotation based on public databases (including SwissProt, NR, KEGG, KOG and Gene Ontology) estimated that 23,017 (90.48%) genes could be classified by at least one of the databases (Fig. 3). In addition, we annotated four types of non-coding RNAs in the *T. crocea* assembly, including micro RNA (miRNA), transfer RNA (tRNA), ribosomal RNA (rRNA), and small nuclear RNA (snRNA). The tRNA genes were predicted by an improved tool for tRNA detection, tRNAscan-SE (version 1.3.1)^45^ with default paramerters. The rRNA fragments were predicted by aligning to invertebrate template rRNA sequences using BlastN (version 2.2.24) at an E-value of 1e-5. The snRNAs as well as miRNAs were identified using INFERNAL (version 1.1.1)^46^ to search against the Rfam database (release 12.0). A total of 1,963 non-coding RNA (ncRNA) were determined in the *Tridacna crocea* genome, including 62 micro RNA (miRNA), 58 small nuclear RNA (snRNA), 83 ribosomal RNA (rRNA), and 1,760 transfer RNA (tRNA) (Table 6).

**Table 6:** Non-coding RNAs in the *Tridacna crocea* assembly.

Type	Copy number	Average length (bp)	Total length (bp)	% of genome
miRNA	62	85	5,269	0.0004
tRNA	1,760	75	131,548	0.0101
rRNA	83	93	7,681	0.0006
snRNA	58	165	9,549	0.0007

Note: ‘% of genome’ was calculated by the non-gap genome size 1,303,216,875 bp.

### Comparative genomic and phylogenetic analysis

We clustered the protein-coding genes into gene families for *T. crocea Aplysia californica (GCF_000002075.1), Crassostrea gigas (GCF_902806645.1), Crassostrea virginica (GCF_002022765.2), Helobdella robusta (GCF_000326865.1), Lottia gigantean (GCF_000327385.1), Mizuhopecten yessoensis (GCF_002113885.1), Octopus bimaculoides (GCF_001194135.1), Drosophila melanogaster (GCF_000001215.4), Homo sapiens (GCF_000001405.39) and Nematostella vectensis (GCF_000209225.1)* (Table 7). 27,422 gene families were identified, of which 3,109 were shared by all eleven species. Comparing with other ten species, there are 347 specific gene families in the *T. crocea* assembly (Fig. 3), among these *T. crocea* specific families, 953 genes are supported by evidence of gene functional annotation. These *T. crocea* specific genes were significantly (P < 0.05) enriched in zinc ion binding, extracellular ligand-gated ion channel activity, integral component of membrane, ion transport related gene ontology (GO) categories (Table 8).

**Table 7:** Statistic analysis of gene families.

Species	Genes number	Genes in families	Unclustered genes	Family number	Unique families	Average genes per family
*T. crocea*	25,440	22,677	2,763	13,000	1,174	1.74
*A. californica*	19,425	12,598	6,827	9,569	334	1.32
*C. gigas*	31,371	28,186	3,185	17,228	859	1.64
*C. virginica*	34,608	31,521	3,087	16,791	936	1.88
*H. robusta*	23,426	15,327	8,099	7,951	685	1.93
*L. gigantea*	23,818	18,798	5,020	12,122	691	1.55
*M. yessoensis*	24,532	20,468	4,064	13,849	749	1.48
*O. bimaculoides*	15,842	12,662	3,180	9,611	290	1.32
*D. melanogaster*	13,972	10,127	3,845	6,791	587	1.49
*H. sapiens*	23,358	19,976	3,382	9,345	980	2.14
*N. vectensis*	23,845	17,653	6,192	9,497	1,132	1.86

Note: Unclustered genes refer to special gene of corresponding species; Unique families refer to special gene families of corresponding species.

**Table 8:** GO enrichment of positive selection genes in *Tridacna crocea*.

GO ID	Involved gene number	Qvalue	GO description
GO:0003723	3	0.047529	RNA binding
GO:0005515	12	0.147774	protein binding
GO:0003824	3	0.253421	catalytic activity
GO:0003676	4	0.376163	nucleic acid binding
GO:0005524	4	0.376163	ATP binding
GO:0016021	6	0.502908	integral component of membrane

**Fig. 4:**
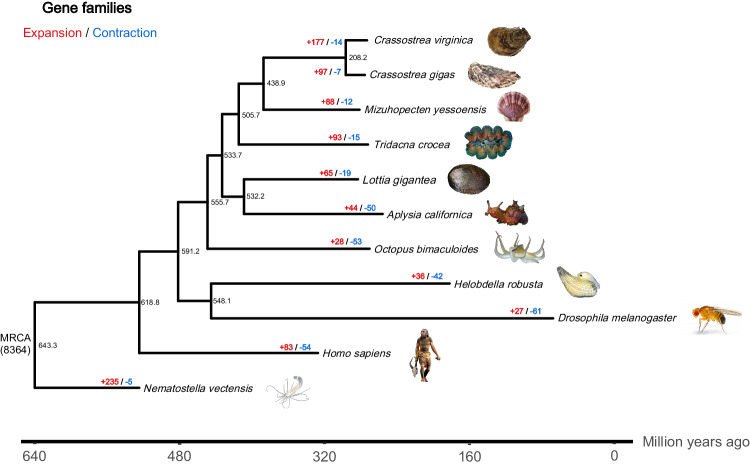
Phylogenetic tree with history of orthologous gene families and divergence times. Numbers on the nodes represent divergence times; parentheses represent error range; MRCA represents most recent common ancestor.

A phylogenetic tree was constructed using the eleven animal species (Fig. 4). Protein sequences were extracted from each family and concatenated to form one supergene for each species, and the maximum likelihood method^47^ was used to reconstruct the phylogenetic tree. The divergence time among the eleven animals was estimated using the MCMCtree program (version 4.4) as implemented in the Phylogenetic Analysis of Maximum Likelihood (PAML) package^48^, with a correlated rates clock and JC69 nucleotide substitution model. The divergence time between *T. crocea* and *M. yessoensis* was predicted to be about 505.7 million years ago (MYA). Compared with the common ancestor of *T. crocea*, *M. yessoensis, C. gigas* and *C. virginica*, *Tridacna crocea* shows 93 and 15 events of gene family expansion and gene family contraction, respectively. The expanded genes in *T. crocea* are related with “DNA replication” (GO:0006260), “DNA-directed DNA polymerase activity” (GO:0003887), “nucleotide binding” (GO:0000166), “methyltransferase activity” (GO:0008168), and so on. On the other side, the contracted genes in the *T. crocea* were significantly (P < 0.05) enriched in GO terms for “iron ion binding” (GO:0005506), “heme binding” (GO:0020037), “oxidoreductase activity, acting on paired donors, with incorporation or reduction of molecular oxygen” (GO:0016705), and “oxidation-reduction process” (GO:0055114).

## Data Records

The raw Illumina, PacBio, Hi-C sequencing and full length transcriptome data are deposited in the NCBI SRA database under the accession numbers SRR17137644^49^, SRR17137645^50^, SRR17137643^51^, and SRR25651021^52^, respectively. The genome assembly and annotations are available from the Figshare^53,54^ and the assembly genome are also deposited at the NCBI with accession number GCA_032873355.1^55^.

## Technical Validation

### Evaluation of the genome assembly

The Hi-C heatmap exhibits the accuracy of genome assembly, with relatively independent Hi-C signals observed between the 18 pseudo-chromosomes (Fig. 1B). To evaluate the quality of the genome assembly, the completeness of the genome assembly was assessed using the conserved metazoan gene set “metazoan_odb10” from the Benchmarking Universal Single-Copy Orthologs (BUSCO) v4.054. The genome assembly was found to have a high level of completeness (94.2%). 74.2% were complete and single-copy, 20% complete and duplicated, 0.6% fragmented, and 5.2% were missing (Table 9). This demonstrates the remarkable completeness and conservation of gene content in giant clam genome assembly, achieving one of the best BUSCO scores observed among reported mollusks. Therefore, these results suggested that the quality of this genome assembly is high.

**Table 9:** Statistic of the *Tridacna crocea* assembly gene-space with the 978 BUSCO metazoa gene set.

	*Tridacna crocea*
Complete BUSCOs*(%)	94.2
Single copy (%)	74.2
Duplicated copy(%)	20
Fragmented (%)	0.6
Missing (%)	5.2

### Genome annotation and phylogenetic analysis

By comparing with public databases including Gene Ontology, KOG, SwissProt, KEGG and NR, gene function information, motifs and domains of their proteins were assigned (Table 10). InterProScan program^56^ with default parameters was used to identify the GO terms and putative domains of genes. For other four databases, the EvidenceModeler-integrated protein sequences against the 4 public protein database were compared using BLASTp^57^ with an E value cutoff of 1e^−05^. Results from the five database searches were concatenated.

**Table 10:** Functional annotation of the predicted genes in the assembly of *Tridacna crocea*.

Type	Gene number	Percentage(%)
Nr	22,337	87.8
Swissprot	19,339	76.02
KEGG	13,008	51.13
KOG	15,425	60.63
GO	14,781	58.1
Annotated	23,017	90.48
Total	25,440	100

The maximum likelihood method was performed to reconstruct the phylogenetic tree according to^47^. The divergence time among the eleven animals were predicted by the MCMCtree program (version 4.4) of Phylogenetic Analysis of Maximum Likelihood (PAML) package^48^, with a correlated rates clock and JC69 nucleotide substitution model. The TimeTree database was used to predict the calibration times of divergence between *Octopus bimaculoides* and *Crassostrea gigas* (~554MYA)^58^.

### Supplementary information

Fig S1

## Data Availability

All data processing commands and pipelines are executed according to instructions and guidelines provided by relevant bioinformatics software. No custom scripts or code were used in this study.
