# Supplementary figures and images for: Chromosome-level genome assembly and annotation of rare and endangered tropical bivalve, Tridacna crocea
Source: Sci Data. 2024 Feb 10;11:186. doi: 10.1038/s41597-024-03014-8 (PMC10858879; doi:10.1038/s41597-024-03014-8)

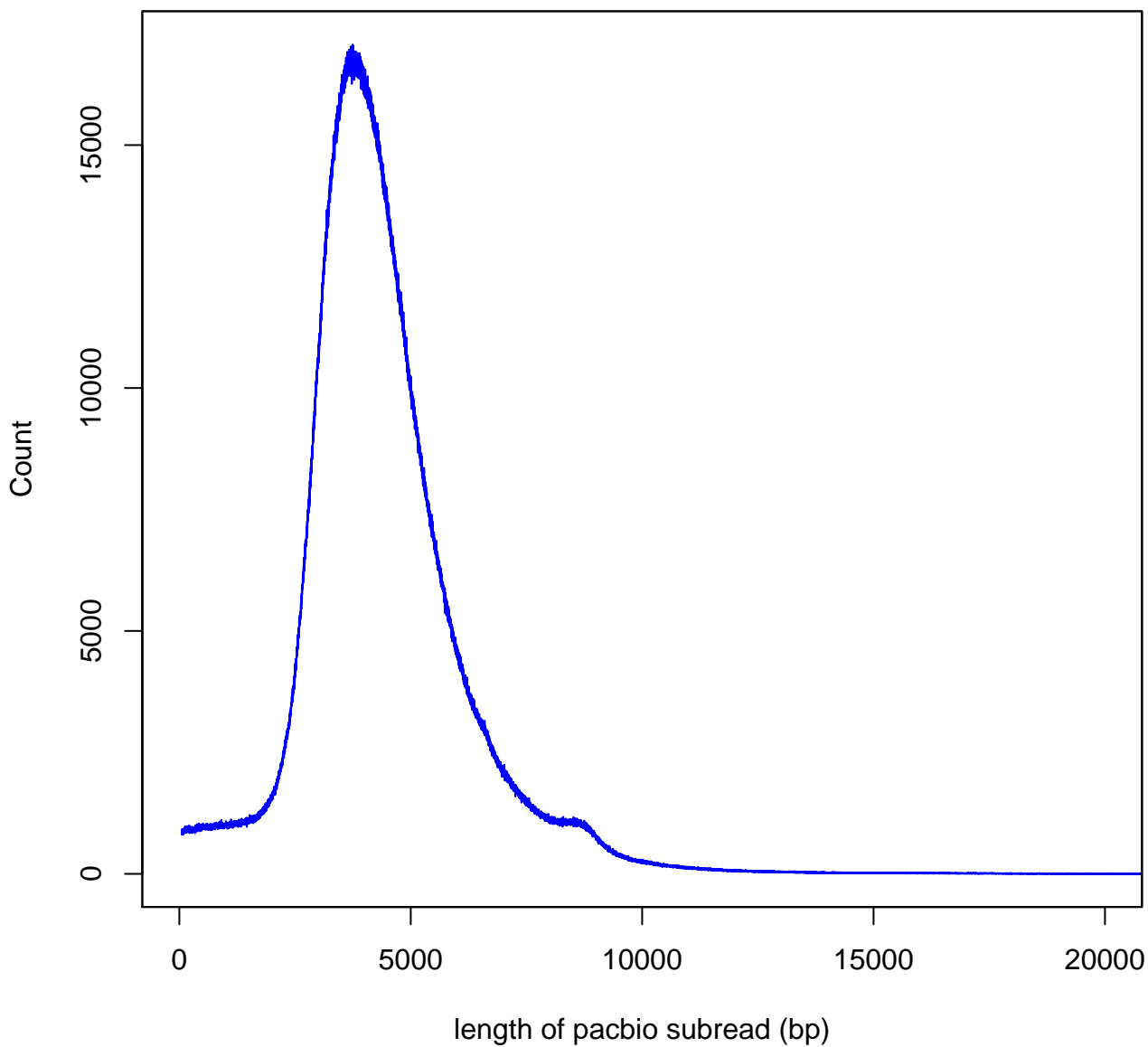

Supplement: Supplementary file 1 — Fig S1 [file 41597_2024_3014_MOESM1_ESM.pdf]
